# Genome-Wide Analysis of miRNA Signature Differentially Expressed in Doxorubicin-Resistant and Parental Human Hepatocellular Carcinoma Cell Lines

**DOI:** 10.1371/journal.pone.0054111

**Published:** 2013-01-24

**Authors:** Jufeng Zhang, Ying Wang, Pingping Zhen, Xia Luo, Chao Zhang, Lin Zhou, Yanxin Lu, Yang Yang, Wei Zhang, Jun Wan

**Affiliations:** 1 Biomedical Research Institute, Shenzhen-PKU-HKUST Medical Center, Shenzhen, China; 2 School of Life Science, Guangdong Pharmaceutical University, Guangzhou, China; 3 Key Lab of Animal Ecology and Conservation Biology, Institute of Zoology, Chinese Academy of Sciences, Beijing, China; 4 College of Engineering, South China Agricultural University, Guangzhou, China; 5 Division of Life Science, Hong Kong University of Science and Technology, Hong Kong, China; Karolinska Institutet, Sweden

## Abstract

Chemotherapy regiments have been widely used in the treatment of a variety of human malignancies including hepatocellular carcinoma (HCC). A major cause of failure in chemotherapy is drug resistance of cancer cells. Resistance to doxorubicin (DOX) is a common and representative obstacle to treat cancer effectively. Individual microRNA (miRNA) has been introduced in the evolution of DOX resistance in HCC in recent studies. However, a global and systematic assessment of the miRNA expression profiles contributing to DOX resistance is still lacking. In the present study, we applied high-throughput Illumina sequencing to comprehensively characterize miRNA expression profiles in both human HCC cell line (HepG2) and its DOX-resistant counterpart (HepG2/DOX). A total of 269 known miRNAs were significantly differentially expressed, of which 23 were up-regulated and 246 were down-regulated in HepG2/DOX cells, indicating that part of them might be involved in the development of DOX resistance. In addition, we have identified 9 and 13 novel miRNAs up- and down-expressed significantly in HepG2/DOX cells, respectively. miRNA profiling was then validated by quantitative real-time PCR for selected miRNAs, including 22 known miRNAs and 6 novel miRNAs. Furthermore, we predicted the putative target genes for the deregulated miRNAs in the samples. Function annotation implied that these selected miRNAs affected many target genes mainly involved in MAPK signaling pathway. This study provides us a general description of miRNA expression profiling, which is helpful to find potential miRNAs for adjunct treatment to overcome DOX resistance in future HCC chemotherapy.

## Introduction

Hepatocellular carcinoma (HCC) is one of the most common virus-associated cancers resulting in high mortality worldwide [1]. For some patients who are not appropriate for surgical treatments, one has to only rely on chemotherapy. However, the development of drug resistance towards chemotherapeutic agents often prevents the successful long-term use of chemotherapy for HCC. Drug resistance, whether intrinsic or acquired over time, becomes the main cause of clinical treatment failure. Therefore, reversing drug resistance has become an emergent issue in tumor treatment. Drug resistance is a multifactorial phenomenon involving many mechanisms, including gene mutation, DNA methylation, altered metabolism and disposition of drugs, altered quantity or activity of target proteins and so on [2]–[6]. Unfortunately, the key underlying mechanisms of the acquisition of resistance to chemotherapeutic agents still remain largely unexplored [2], [7].

MiRNAs are endogenously expressed small non-coding RNAs of 18–25 nucleotides in length that can post-transcriptionally regulate gene expression across various biological processes [8]–[12]. Growing evidences have revealed that miRNAs are important regulators in many signaling pathways involved in tumor pathogenesis [8], [9], [13]. MiRNA signature has been identified as pathological markers for HCC diagnostic discriminator and predictive prognosis of patients [2], [14], [15]. They might serve as tumor suppressors or oncogenes and constitute ideal targets in exploring anticancer therapeutics [6], [16], [17]. Besides, overwhelming efforts have been exerted in analyzing the role of miRNAs in the development of drug resistance in tumor cells. Blower et al. and Liu et al. carried out systematical studies respectively to explore the correlation between miRNA expression levels and drug activity across the NCI-60 cell lines (HepG2 not included) [18], [19]. Tomimaru and colleagues reported that miRNA-21 is a significant modulator of the anti-tumor effect of interferon (IFN)-α/5-fluorouracil (5-FU) using quantitative real-time RT-PCR (qRT-PCR) in both HCC cell lines and clinical HCC samples [20]. Tomokuni et al. performed miRNA microarray analysis and found that miRNA-146a regulated the sensitivity of HCC cells to IFN-α [21]. Another group recently identified the role of miRNA-195 in developing drug resistance in HCC cell line using qRT-PCR [22]. They found that miRNA-195 might improve 5-FU sensitivity by targeting Bcl-w protein to increase cell apoptosis. Current studies have, thus far, been conducted to support the hypothesis that up- or down-expression of a certain miRNA can be tied to a patient's response to anti-cancer drugs [6]. Among huge amounts of chemotherapeutics, doxorubicin (DOX) is one of the most used and front line drugs for treating patients with HCC. Down-regulation of miRNA-122 was found to contribute to hepatocarcinogenesis and DOX challenge by targeting cyclin G1 and modulating p53 pathway [23]. Restoring attenuated levels of miRNA-199a could increase sensitivity to DOX-induced apoptosis [24]. Apparently, most previous studies attempting to detect miRNA signature relevant to cancer chemosensitivity and chemoresistance have scanned only known individual miRNAs. Since many miRNAs are reported to be involved in the development of drug resistance in HCC, it is becoming important to use global and systematic analytic techniques to assess the miRNA expression profiles. Identification of miRNAs in drug resistant HCC cells and their parental ones by deep sequencing technology may provide a quantitative analysis of known individual miRNA and the possibility to discover novel miRNAs.

In this study, comprehensive expression profiling of miRNAs by deep sequencing was performed in DOX-resistant and parental HCC cells. We identified a panel of differentially expressed known and novel miRNAs, which contribute to better understanding of miRNAs' roles in the formation of drug resistance in HCC cells.

## Materials and Methods

### Cell culture

The human hepatocarcinoma cell line HepG2 was obtained from Cell Bank of Chinese Academy of Sciences (Shanghai, China) and maintained in Dulbecco's modified Eagle's medium (DMEM) containing 10% fetal bovine serum (FBS), 2 mM glutamine, 100 U/ml penicillin and 100 µg/ml streptomycin at 37°C in a humidified atmosphere of 5% CO_2_. The DOX-resistant variant of HepG2 cells (HepG2/DOX) was established by continuous culture in medium containing stepwise increasing concentration of DOX at a range of 0.5 to 25 µM over a period of 10 months. After 10 months of culturing in the presence of DOX, the 50% inhibitory concentration (IC_50_) values were 23 and 0.6 µM DOX for the HepG2/DOX and parental HepG2 cells, respectively.

### RNA extraction

Total RNA was extracted from HepG2 and HepG2/DOX using TRIZOL (Invitrogen, US) in accordance with the manufacture's protocol. RNA samples then passed the RNA quality control for deep sequencing.

### Small RNA library construction and sequencing

Small RNA library construction and sequencing were conducted as previously described [25], [26]. Briefly, small RNAs with 18–30 nt in length were first isolated from total RNA through size fractionation using 15% tris-borate-EDTA (TBE) urea polyacrylamide gel. The separated small RNAs were then ligated to 5′ adaptor (5′- GUUCAGAGUUCUACAGUCCGACGAUC) and 3′ adaptor (5′-UCGUAUGCCGUCUUCUGCUUGUidT). The ligated RNAs were size fractionated on a 15% TBE urea polyacrylamide gel again and then the excised RNAs with 5′ and 3′ adaptors were reversely transcribed to cDNA with the RT primer (CAAGCAGAAGACGGCATACGA). The cDNA was taken as a template for PCR amplification using primer set (5′-CAAGCAGAAGACGGCATACGA-3′; 5′-AATGATACGGCGACCACCGACAGGTTCAGAGTTCTACAGTCCGA-3′). After purification and quantification, the resulting PCR products were sequenced on the Illumina Cluster Station and Genome Analyzer II (Illumina Inc, USA) following the manufacturer's protocol.

### Sequencing data analysis process

The 50-nt sequence reads yielded by deep sequencing passed through data cleaning process, including getting rid of the low quality reads and several kinds of adaptor-adaptor contaminants. The occurrences of each unique sequence reads were counted as tags. Normally, miRNA is enriched around 21 nt or 22 nt, siRNA is 24 nt, and piRNA is 30 nt. Here only length of small RNAs between 18 nt and 30 nt were retained for further analyses. The standard bioinformatics analysis to annotate the clean tags was as follows: (1) Map all the small RNA tags that pass filters to the reference human genome by Short Oligonucleotide Alignment Program (SOAP 2.0) and analyze their expression and distribution on the genome [27]; (2) Annotate the small RNA tags with rRNA, scRNA, snoRNA, snRNA and tRNA using Rfam 10.1 (http://rfam.sanger.ac.uk/) and Genbank (http://www.ncbi.nlm.nih.gov/genbank/) databases to get rid of matched tags from unannotated tags; (3) Align the small RNA tags to the miRNA precursor/mature miRNA of human species in miRBase18 (http://www.mirbase.org/) [28] to get (a) the known miRNA count, (b) base bias on the first position among identified miRNAs with fixed length (18–30 nt), (c) base bias on each position among all identified miRNAs; (4) Align the small RNA tags to repeated associated RNA to find matched tags in the samples; (5) Align the small RNA tags to exons and introns of mRNA and match the small RNA to their original sites in genome; (6) If the clean tags can not be annotated to any category, we took them to predict the novel miRNAs. To make every specific small RNA mapped to only one annotation, we obeyed the following priority rule: rRNA etc (in which Genbank>Rfam)>known miRNA>repeat>exon>intron [29]. The total rRNA ratio of less than 40% was a mark for sample quality check.

Mireap (http://sourceforge.net/projects/mireap) [25] was developed to predict novel miRNA candidates based on their secondary hairpin structure, the Dicer cleavage site and the minimum free energy of the unannotated small RNA tags. In general, the prediction accuracy could be assessed according to the following two criteria for defining high-confidence miRNA candidates: (1) the characteristic stable hairpin structure with low free energy (<−20 kcal/mol); (2) expressed in both two samples at detectable levels (1 TPM, one transcript per million tags) [26].

### Detection of differentially expressed miRNAs and prediction of target genes

Detection of differentially expressed miRNAs between HepG2 and HepG2/DOX cells was similar as described previously [25]. If the adjusted *P*-values were <0.05 based on the Benjamini and Hochberg multiple testing correction [30], [31] and there was at least a 2-fold change ((HepG2/DOX)/HepG2) in the normalized expression level, one could consider the miRNAs as significantly differentially expressed. The target genes for each differentially expressed miRNA were mainly predicted by Mireap (http://sourceforge.net/projects/mireap) [25]. Given that the prediction softwares often suffer from high false positive rates, we used other three tools to assist the prediction, including miRanda (http://www.microrna.org/microrna/home.do) [32], PicTar (http://pictar.mdc-berlin.de/) [33] and miRDB (http://mirdb.org/miRDB/) [34], [35]. Only the target genes predicted by at least three independent tools were taken into account. The Gene Ontology (GO) [36] terms and Kyoto Encyclopedia of Genes and Genomes (KEGG ) [37] pathways of target genes were annotated.

### Validation of miRNA expression by quantitative RT-PCR

Assays to quantify the known and novel miRNAs were done by using miScript PCR System (Qiagen) according to the manufacturer's instruction. RT reactions with miScript II RT Kit (Qiagen) contained 1.0 µg total RNA, 4 µl 5×miScript HiSpec buffer, 2 µl 10×miScript nucleics mix and 2 µl miScript reverse transcriptase mix in each reaction (20 µl). The RT reaction was conducted under the following conditions: 37°C for 60 min and then 95°C for 5 min. After that, the cDNA products from RT reaction were diluted 15 times. PCR was carried out with 1.5 µl of the diluted products in 20 µl PCR reaction containing 10 µl 2×QuantiTect SYBR Green PCR master mix, 2 µl 10×miScript universal primer, 2 µl 10×miScript primer assay. Amplification was performed as follows: 95°C for 15 min, followed by 40 cycles at 94°C for 15 s, 55°C for 30 s and 70°C for 30 s. All reactions were run in triplicate. Relative expression was calculated using the comparative CT method and normalized to the expression of RNU6B.

## Results

### Sequencing data description

For each sample, we obtained ∼14 M clean reads from the raw sequences (Table S1, data available in http://www.ncbi.nlm.nih.gov/Traces/sra/, submission number: SRA060665). The quite equal total number of reads between HepG2 and HepG2/DOX cells will allow a reliable comparison of miRNA distribution and expression profiles in the following steps. To assess the size distribution of small RNAs in each library, we counted the number of clean reads based on the insert length. The most abundant group in both samples was 22 nt in length, as most studies of miRNA size distribution reported in human or animals. Two samples presented different patterns of distribution: tags with 22 nt comprised ∼64.00% of the total number of small RNAs in HepG2, while only ∼25.00% in HepG2/DOX small RNA pools (Figure S1). The lower distribution of 22-nt tags in the latter sample might indicate that most miRNAs were down-expressed in HepG2/DOX.

We then summarize the common and specific tags between two samples, including the summary of unique tags and total tags. In both HepG2 and HepG2/DOX cells, we obtained 932,661 unique tags after removing repeats from the total tags (27,733,155). The two samples shared 6.64% and 89.07% of unique common tags and total common tags. The very few unique common tags indicated that HepG2/DOX presented a distinctive small RNA profile compared to HepG2. HepG2/DOX had more specific small RNAs than HepG2 (unique: 62.40% and 30.96%; total: 8.12% and 2.81% for HepG2/DOX and HepG2, respectively) (Figure S2).

In HepG2 and HepG2/DOX, most 22-nt small RNAs began with the base “U”. The 21-nt small RNAs exhibited a bias to “A” and “U” at first base in HepG2, but only “A” in HepG2/DOX (Figure S3). Both libraries displayed similar compositions of four bases and most sites possessed a major base, which indicated that small RNA sequences from libraries were conserved.

### Mapping

**Figure 1:**
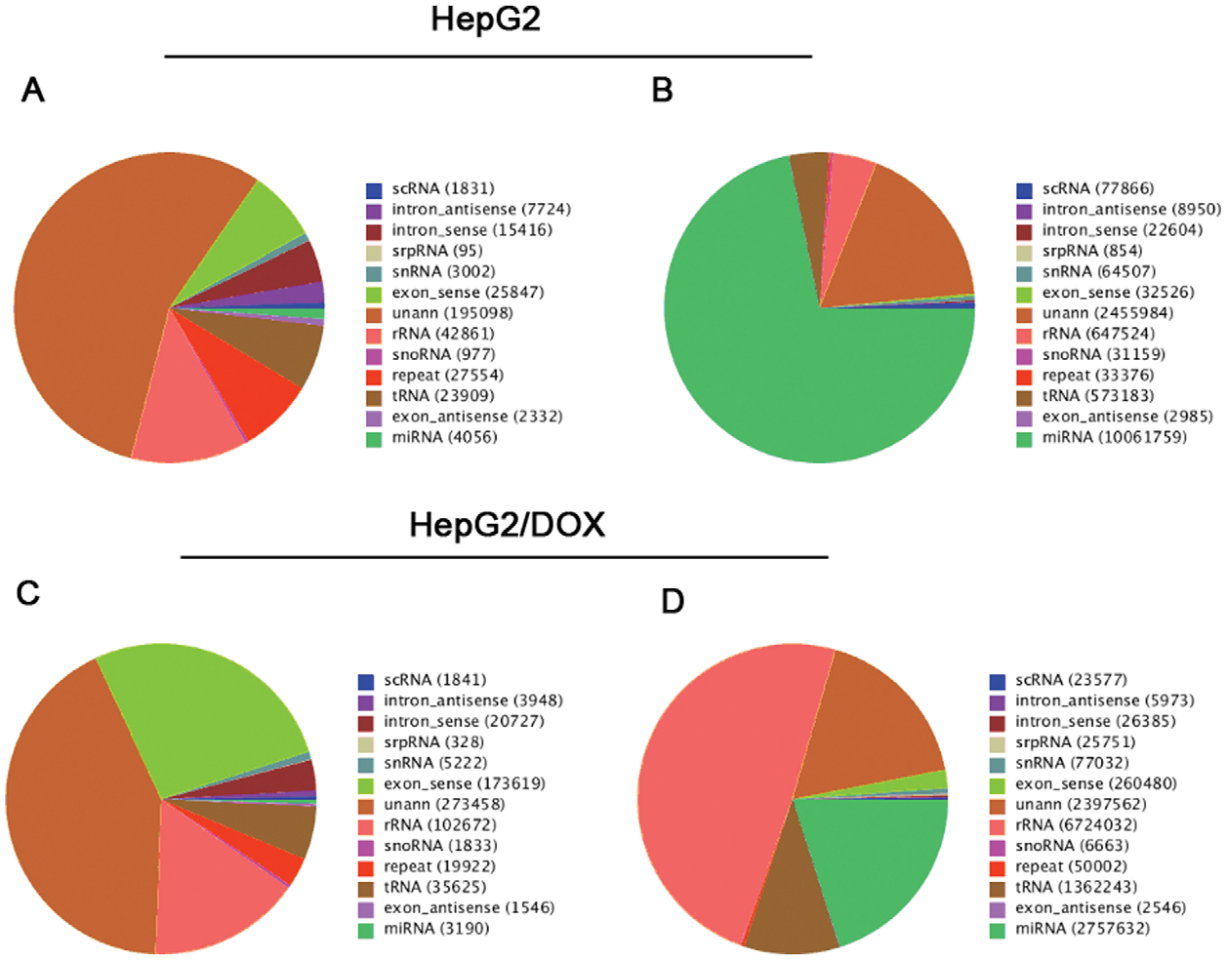
Annotation and distribution of small RNAs among different categories. (A) Pie chart for annotation of unique tags of small RNAs in HepG2; (B) Pie chart for annotation of total tags of small RNAs in HepG2; (C) Pie chart for annotation of unique tags of small RNAs in HepG2/DOX; (D) Pie chart for annotation of total tags of small RNAs in HepG2/DOX.

With regard to the unique tags, a total of 42.10% (147,647 tags) from HepG2 and 47.79% (307,759 tags) from HepG2/DOX were mapped onto human genomes. Concerning the total tags, about 78.73% (11,033,112 tags) from HepG2 and 73.82% (10,128,686 tags) from HepG2/DOX were mapped onto human genomes. As shown in Figure S4, small RNAs were unevenly distributed across chromosomes between sense and antisense chains. More small RNAs were mapped in the sense chains. For example, there were 25,847 unique tags and 2,332 unique tags in the exon-sense and exon-antisense regions in HepG2, respectively, with ratio of 11∶1; while in HepG2/DOX, the ratio was 112∶1 (173,619∶1,546) (Figure 1A and 1C). As shown in Figure 1B and 1D, the numbers of total tags were 32,526 and 2,985 on the exon-sense and exon-antisense chains in HepG2, while 260,480 and 2,546 in HepG2/DOX.

### Categorization and annotation

We categorized the small RNA tags into miRNA, scRNA, tRNA, rRNA, snRNA/snoRNA, repeats, and mRNA fragments. From the annotated small RNAs in Genbank and Rfam (10.1) databases, we obtained 4,056 and 3,190 miRNAs in HepG2 and HepG2/DOX cells, respectively (Figure 1). There were still 55.63% and 42.47% unannotated small RNAs in two samples, which were used for novel miRNA prediction. As for known miRNAs, we referred to the database of miRBase18 and detected 780 mature miRNA (including 270 miRNA-5p and 246 miRNA-3p) and 680 miRNA precursors from HepG2,and 668 mature miRNA (including 231 miRNA-5p and 209 miRNA-3p) and 585 miRNA precursors from HepG2/DOX (Table S2). In total, we got 360 miRNAs shared by both samples, whose expression levels were then normalized and compared.

### Differentially expressed known and novel miRNAs between HepG2 and HepG2/DOX cells

**Figure 2:**
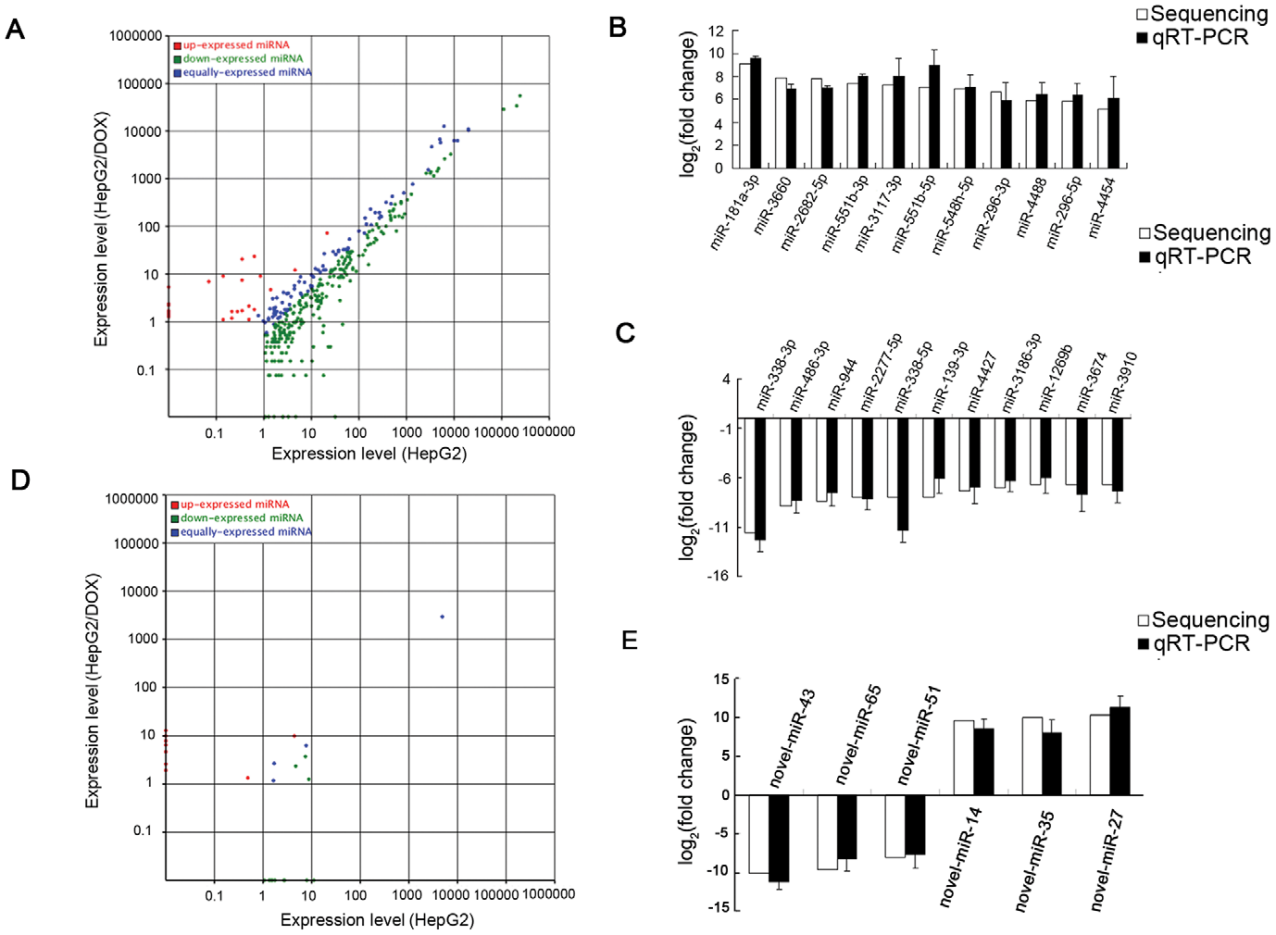
The miRNA expression levels from deep sequencing and validation with qRT-PCR. (A) The scatter plot shows the expression levels of known miRNAs in HepG2 and HepG2/DOX cells. Blue dots: equally expressed miRNAs between HepG2 and HepG2/DOX; Red dots: miRNAs in HepG2/DOX were up-expressed compared to HepG2 (adjusted *P*<0.05); Green dots: miRNAs in HepG2/DOX were down-expressed compared to HepG2 (adjusted *P*<0.05). (B,C) The validation of selected up- and down-expressed known miRNAs indicated that the results from deep sequencing were generally agreed well with the qRT-PCR results. (D) The scatter plot shows the expression levels of novel miRNAs in HepG2 and HepG2/DOX cells. (E) The validation of selected novel miRNAs indicated that the results from deep sequencing were generally agreed well with the qRT-PCR results.

In all the 360 known miRNAs shared by both cells, 23 miRNAs were over-expressed and 246 miRNAs were down-expressed significantly (adjusted *P*<0.05) in the HepG2/DOX cells (Figure 2A). Among these differentially expressed miRNAs, HepG2/DOX cells had a total of 12 miRNAs with elevated expression levels >16-fold and 30 miRNAs with reduced expression levels >16-fold of the corresponding miRNAs in HepG2 cells (Table 1).

**Table 1:** The most significantly differentially expressed miRNAs in HepG2/DOX cells.

miRNA ID	log_2_(fold change)*	Up/down expression	*P*- value adjusted	miRNA ID	log_2_(fold change)*	Up/down expression	*P*- value adjusted
hsa-miR-181a-3p	9.04	Up	1.56E-23	hsa-miR-338-3p	−11.63	Down	4.47E-133
hsa-miR-3660	7.87	Up	1.95E-11	hsa-miR-486-3p	−8.90	Down	2.14E-21
hsa-miR-2682-5p	7.77	Up	7.78E-11	hsa-miR-944	−8.39	Down	1.61E-15
hsa-miR-551b-3p	7.39	Up	9.63E-09	hsa-miR-2277-5p	−8.01	Down	2.67E-12
hsa-miR-3117-3p	7.26	Up	3.73E-08	hsa-miR-338-5p	−8.01	Down	2.68E-12
hsa-miR-551b-5p	7.11	Up	1.49E-07	hsa-miR-139-3p	−7.96	Down	9.21E-75
hsa-miR-548h-5p	6.95	Up	5.93E-07	hsa-miR-4427	−7.36	Down	1.58E-08
hsa-miR-296-3p	6.60	Up	8.05E-29	hsa-miR-3186-3p	−7.01	Down	4.40E-07
hsa-miR-4488	5.95	Up	4.10E-35	hsa-miR-1269b	−6.74	Down	3.15E-06
hsa-miR-296-5p	5.84	Up	3.88E-78	hsa-miR-3674	−6.74	Down	3.16E-06
hsa-miR-4454	5.19	Up	3.07E-85	hsa-miR-3910	−6.74	Down	3.17E-06
hsa-miR-4687-3p	4.37	Up	8.32E-26	hsa-miR-642a-3p	−6.74	Down	5.10E-32
hsa-miR-3654	3.38	Up	1.21E-25	hsa-miR-486-5p	−6.46	Down	2.81E-100
hsa-miR-577	2.94	Up	8.64E-05	hsa-miR-203	−6.30	Down	3.19E-89
hsa-miR-4508	2.9	Up	5.94E-06	hsa-miR-1277-3p	−6.16	Down	3.01E-41
hsa-miR-3687	2.49	Up	2.04E-05	hsa-miR-1277-5p	−5.90	Down	3.84E-18
hsa-miR-4448	2.45	Up	1.66E-04	hsa-miR-1250	−5.71	Down	2.05E-169
hsa-miR-4426	2.23	Up	3.53E-05	hsa-miR-139-5p	−5.46	Down	3.71E-25
hsa-miR-720	2.08	Up	1.21E-05	hsa-miR-335-5p	−5.26	Down	2.00E-41
hsa-miR-4485	1.71	Up	6.14E-08	hsa-miR-362-3p	−5.01	Down	3.36E-10
hsa-miR-877-5p	1.68	Up	1.92E-82	hsa-miR-1255a	−4.92	Down	1.25E-62
hsa-miR-3679-5p	1.45	Up	5.53E-04	hsa-miR-3622a-5p	−4.83	Down	4.30E-09
hsa-miR-21-3p	1.40	Up	7.87E-13	hsa-miR-1293	−4.67	Down	2.87E-08
				hsa-miR-2116-3p	−4.64	Down	3.67E-14
				hsa-miR-365a-3p	−4.52	Down	8.20E-62
				hsa-miR-365b-3p	−4.52	Down	8.29E-62
				hsa-miR-4501	−4.22	Down	2.27E-06
				hsa-miR-196a-3p	−4.14	Down	4.15E-06
				hsa-miR-500a-5p	−4.01	Down	2.94E-09
				hsa-miR-500b	−4.01	Down	2.94E-09

Note: *fold change = (HepG2/DOX)/HepG2.

To further validate these differentially expressed known miRNAs, 22 individual miRNAs were selected to perform quantitative RT-PCR assay. These 22 selected miRNAs covered both top up-expressed miRNAs (miR-181a-3p, miR-3660, miR-2682-5p, miR-551b-3p, miR-3117-3p, miR-551b-5p, miR-548h-5p, miR-296-3p, miR-4488, miR-296-5p, miR-4454) and top down-expressed miRNAs (miR-338-3p, miR-486-3p, miR-944, miR-2277-5p, miR-338-5p, miR-139-3p, miR-4427, miR-3186-3p, miR-1269b, miR-3674, miR-3910) in HepG2/DOX cells. As illustrated in Figure 2B and 2C, the Illumina deep sequencing data correlated well with the quantitative RT-PCR results, indicating the reliability of sequencing based expression analysis.

To discover novel candidate miRNAs in HepG2 and HepG2/DOX cells, we predicted the sequences with miRNA stem loop structure and Dicer cleavage sites from unannotated small RNA sequences to be novel miRNAs. A total of 71,596 and 41,059 sequence tags were identified to be 70 and 59 novel miRNAs in HepG2 and HepG2/DOX, respectively, of which 26 miRNAs were shared by both cells. Compared to HepG2 cells, 9 and 13 novel miRNAs were significantly up-expressed and down-expressed in HepG2/DOX cells, respectively (Figure 2D, Table 2, adjusted *P*<0.05). We selected 6 novel miRNAs for further validation by quantitative RT-PCR assay, including novel-miR-43, novel-miR-65, novel-miR-51, novel-miR-14, novel-miR-35 and novel-miR-27. As a result, the above predicted miRNA candidates could be successfully amplified by quantitative RT-PCR and the level of expression coincided with the sequencing results (Figure 2E). These differentially expressed miRNAs or their combined expression might regulate mRNAs associated with the DOX resistance of HCC cells.

**Table 2:** Significantly differentially expressed novel miRNAs in HepG2/DOX cells.

miRNA ID	log_2_(fold change)*	Up/down expRession	*P*- value	miRNA ID	log_2_(fold change)*	Up/down expRession	*P*- value
hsa-novel-mir-27	10.32	Up	7.84E-55	hsa-novel-miR-43	−10.14	Down	3.29E-48
hsa-novelmiR-35	9.95	Up	5.69E-43	hsa-novel-miR-65	−9.644	Down	1.24E-34
hsa-novel-miR-14	9.59	Up	7.59E-34	hsa-novel-miR-51	−8.12	Down	3.59E-13
hsa-novel-mir-8	9.31	Up	4.43E-28	hsa-novel-miR-41	−8.08	Down	7.06E-13
hsa-novel-miR-12	8.84	Up	8.36E-21	hsa-novel-miR-64	−7.48	Down	4.18E-09
hsa-novel-miR-25	7.99	Up	2.48E-12	hsa-novel-miR-50	−7.29	Down	3.06E-08
hsa-novel-mir-15	7.57	Up	1.23E-09	hsa-novel-miR-72	−7.29	Down	3.05E-08
hsa-novel-miR-17	1.39	Up	1.75E-03	hsa-novel-miR-71	−7.23	Down	5.87E-08
hsa-novel-miR-26	1.12	Up	1.58E-08	hsa-novel_miR-58	−7.08	Down	2.25E-07
				hsa-novel-miR-57	−6.74	Down	3.13E-06
				hsa-novel-miR-13	−2.82	Down	1.41E-21
				hsa-novel-miR-19	−1.04	Down	4.77E-05
				hsa-novel-miR-5	−1.01	Down	2.18E-06

Note: *fold-change = (HepG2/DOX)/HepG2.

### Prediction of miRNA target genes

**Figure 3:**
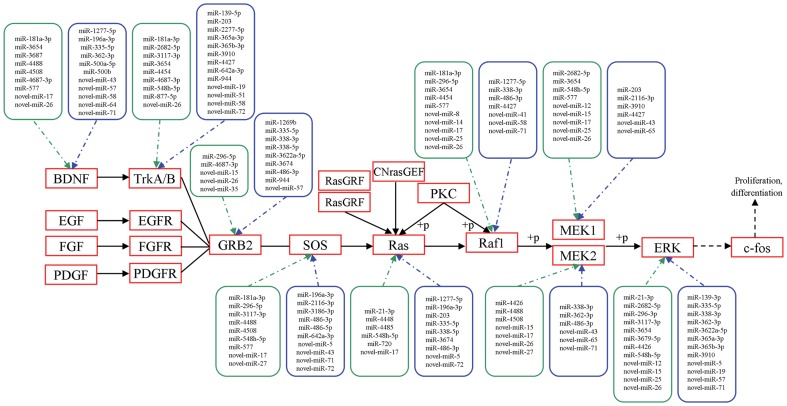
Differentially expressed miRNA-modulated putative targets in the part of MAPK signaling pathway. Kyoto Encyclopedia of Genes and Genomes (KEGG) analysis was conducted to identify some of the putative targets for the most differentially expressed known and novel miRNAs in the MAPK signaling pathway. Here only part of the pathway was displayed. miRNAs shown in green box are up-regulated while those in blue box were down-regulated.

We predicted the potential target genes of aberrantly expressed miRNAs in HepG2/DOX cells. Based on prediction of Mireap software, these miRNAs were expected to target 31,175 genes in total. From the results, we observed that a single miRNA could influence thousands of potential targets (e.g. the most up- and down- expressed miRNAs, miRNA-181a-3p and miRNA-338-3p, target 6056 and 11814 genes, respectively) and the same gene could also be targeted by multiple miRNAs (Figure 3).

To explore the biological functions of the most differentially expressed miRNAs (miRNA-181a-3p and miRNA-338-3p), we further checked their potential targets by other three prediction methods: PicTar, miRDB and miRanda. The genes detected by three of four independent tools including Mireap were considered to be the targets of miRNAs. Of the most up-expressed miRNA (miRNA-181a-3p) in HepG2/DOX, one target gene RBM22 supported by three softwares (Mireap, miRDB and PicTar), was expected to be the common targets of seven other significantly differentially expressed miRNAs including miR-21, miR-101, miR-217, miR-590-5p, miR-181b, miR-181c, and miR-181d. Based on GO database, this gene is suggested to participate in cellular response to pressure (GO:0033554), mRNA splicing (GO:0045292, GO:0000398, and GO:0033120), protein translocation and transporting (GO:0090316 and GO:0000060). The target gene of the most down-expressed miRNA (miRNA-338-3p) was UBE2Q1, which was detected by three prediction softwares (Mireap, PicTar and miRanda) and validated by experimental tests as well [38] (http://mirecords.biolead.org). It is also the common candidate target of other ten significantly differentially expressed miRNAs, i.e. miR-203, miR-195-5p, miR-497-5p, miR-424-5p, miR-16, miR-15b-5p, miR-27a, miR-27b, miR-101 and miR-590-3p. This target gene has ubiquitin-protein ligase activity (GO:0004842) and it has been shown to join in the pathways that significantly influence the resistance of chemotherapeutics in HCC therapy [39].

The differentially expressed novel miRNAs were predicted to target 30,240 mRNAs potentially based on Mireap analysis. We further inferred the functions of potential miRNA targets from GO enrichment, including molecular function, cellular component and biological process. Here, we only listed the significant terms belonging to biological process in Table 3 (adjusted *P*<0.05, Bonferroni Correction). As shown in the table, most of the significant GO terms are related to the metabolic process (e.g. GO: 0044238, GO: 0060255 and GO: 0006139). Other GO categories such as regulation of gene expression (GO: 0010468), protein modification process (GO: 0006464) and transcription (GO: 0006351) are also significantly enriched.

**Table 3:** The GO term of predicted targets of differentially expressed novel miRNAs.

GO ID	GO term	Gene count	Percentage (%)	Bonferroni correction
GO:0010468	regulation of gene expression	3697	16.0%	0.00064
GO:0060255	regulation of macromolecule metabolic process	4098	17.8%	0.00073
GO:0006464	protein modification process	3103	13.5%	0.00516
GO:0019222	regulation of metabolic process	5101	22.1%	0.00892
GO:0006351	transcription	3607	15.6%	0.00902
GO:0043412	biopolymer modification	3296	14.3%	0.01392
GO:0044238	primary metabolic process	11875	51.5%	0.01610
GO:0006139	nucleobase,nucleoside, nucleotide and nucleic acid metabolic process	6208	26.9%	0.02706
GO:0050789	regulation of biological process	9522	41.3%	0.03080
GO:0043170	biopolymer metabolic process	9444	41.0%	0.03713

To explore the role that aberrantly expressed miRNAs play in the regulatory networks, we assigned putative targets of novel miRNAs into KEGG pathways. We discovered 6 pathways were enriched, of which the MAPK signaling pathway was the most prominent (adjusted *P*<0.05, Bonferroni Correction, Table 4). Besides, KEGG analysis offered us further information that MAPK was also one of the important pathways that gathered the putative targets for the top up- and down-expressed known miRNAs, including miR-181a-3p (213 genes), miR-3660 (36 genes), miR-2682-5p (67 genes), miR-551b-3p (19 genes), miR-3117-3p (51 genes) and miR-338-3p (327 genes), miR-486-3p (297 genes), miR-944 (30 genes), miR-2277-5p (159 genes), miR-338-5p (189 genes). Among the analyzed miRNAs, some have common target genes in the MAPK signaling pathway that combined to a systematic network (Figure 3). In HCC, MAPK signaling pathways are up-regulated and generally considered to promote tumor growth [26], [40]–[43]. It has been addressed to participate in the drug-induced tumor cell proliferation and apoptosis in other studies [44]–[46].

**Table 4:** The KEGG term of predicted targets of differentially expressed novel miRNAs.

Pathway ID	Pathway	Gene count	Percentage (%)	Bonferroni correction
ko04010	MAPK signaling pathway	772	(3.28%)	0.00101
ko00230	Purine metabolism	1652	(7.02%)	0.00515
ko04621	NOD-like receptor signaling pathway	236	(1%)	0.01924
ko04144	Endocytosis	649	(2.76%)	0.03904
ko04730	Long-term depression	3	(0.82%)	0.03964
ko04971	Gastric acid secretion	550	(2.34%)	0.04692

## Discussion

MiRNAs have recently been shown to play important roles in the development of chemoresistance. Resistance to DOX is a common and representative barrier for successful treatment of cancers. In the present study, we determined the miRNA expression profiles that were differentially expressed between HepG2/DOX and parental HepG2 cells through deep sequencing, which was followed by validation with qRT-PCR. We then carried out function annotation for the predicted target genes of novel miRNAs with GO and KEGG analyses.

The results demonstrated that 23 miRNAs were over-expressed in HepG2/DOX cells, of which miRNA-181a-3p was the most up-regulated. It has been studied in various malignancies showing opposite results. For example, miRNA-181a has been shown to be linked to improved survival in chronic lymphocytic leukemia and acute myelogenous leukemia [47]. It is also indicated that miRNA-181a is an inhibitor of oncogenesis and tumor growth, therefore it can improve prognosis and reduce recurrence risk in gliomas [48]. In hepatocellular cancer cell lines, over- expression of miRNA-181a can diminish adhesion and migration of cancer cells through suppressing glycophosphoprotein OPN expression [49]. In contrast, miRNA-181a expression level has also been reported to be of importance in epithelial cell adhesion molecule^+^/alpha-fetoprotein^+^ (EpCAM^+^/AFP^+^) HCC cells quantity, resulting in increased metastasis and poor survival [50]. In our results, miRNA-181a-3p is a specific miRNA in HepG2/DOX cells after normalization. It might come into play through negative regulation of tumor suppressor genes and/or genes that suppress cell differentiation or apoptosis, which contributes to the development of DOX resistance.

Interestingly, most miRNAs were down-regulated in DOX-resistant cells, accounting for about 91.4% of the total miRNAs we presented here. We found miRNA-338-3p was the most down-regulated miRNA in HepG2/DOX cells. It has been reported that miRNA-338 was down-regulated in surgically removed HCC tissues through bead-based microarray [51]. The same group also showed that miRNA-338-3p suppressed HCC cell invasion by targeting smoothened (SMO) and matrix metalloproteinase (MMP-9) [52]. It is indicated that miRNA-338-3p together with other down-regulated miRNAs might be effective in avoiding resistance or enhancing the effectiveness of HCC to chemotherapy.

Among all the other differentially expressed miRNAs we discovered, quite a lot have been reported by other studies about DOX resistance in HCC. A good example is the down-regulation of miRNA-122 as a liver-specific miRNA in our HepG2/DOX cells [53], [54]. It has been shown that over-expression of miRNA-122 can silence its target cyclin G1 and increase DOX sensitivity of HCC cells [23]. MiRNA-122 was also suggested to sensitize HCC cells to chemotherapeutics through modulating expression of MDR. [55]. MiRNA-199a/b-3p is reported to be down-regulated in HCC and it increases sensitivity to DOX-induced apoptosis by targeting mTOR and c-met. We got the same results with lower expression level of miRNA-199a-3p in HepG2/DOX cells [24], [56]. Besides, we also found that miRNA-296 have a very high expression level in HepG2/DOX cells. This miRNA can suppress p53 function in cancer cells and might contribute to overcome DOX challenge [57].

Pathway analysis revealed that most significantly regulated miRNAs have putative targets in a common pathway, the MAPK pathway. In line with our predicted targets of known and novel miRNAs, multiple components in this critical mitogenic pathway have been reported to favor the development of drug resistance. For example, an enhanced level of phosphorylated p38 is displayed to be responsible for chemotherapy resistance in colon and gastric cancer cells [58], [59]. The TrkB-BDNF interaction is linked to enhanced survival and resistance to DOX, etoposide, and cisplatin in neuroblastoma [60]. The Ras-RAF-MEK-ERK MAP kinase signaling is also taken as a critical target of anti-drug resistance [61]–[63]. Therefore, molecular targeting of this critical mitogenic pathway may represent an alternative way for the treatment of HCC.

Since the discovery of miRNAs' contribution to cancers, several technical platforms have been conducted in elucidating the differential expression of miRNAs, including microarray, qRT-PCR and deep sequencing. Unlike the former approaches focusing on the alteration of individual miRNA, deep sequencing depicts the abundance of each miRNA in the full scale of miRNome. It is one of the most effective and accurate approaches to discriminate the abnormally expressed miRNAs in tumor genomes. To our knowledge, deep sequencing of miRNome related to DOX resistance has never been done in HCC. This direct sequencing offers the possibility to obtain millions of small RNA sequence tags in one shot and detect the length variation of mature miRNA as well as base bias. With benefit of next generation sequencing, we not only displayed the expression differences of modest or even low abundant miRNAs between two samples, but also discovered a number of novel miRNAs.

The contribution of miRNAs in anticancer drug resistance is a very complicated question. The study of linking different miRNAs to various targets and genetic pathways is still in infancy stage. Identification of the differentially expressed miRNAs in human HCC cells related to the resistance of chemotherapy regiments might help to predict the response to chemotherapy. In addition, manipulation of miRNA functions combined with traditional chemotherapy agents might provide a highly promising therapeutic strategy for future treatment of malignant tumors.

## Supporting Information

Figure S1 **Size distributions of small RNAs in HepG2 and HepG2/DOX cells.** 22-nucleotide small RNAs are enriched in both samples, which is in line with the typical size of miRNAs.
(TIF)

Figure S2 **The proportions of unique common tags and total common tags of small RNAs detected in HepG2 and HepG2/DOX cells.** The very few unique common tags indicate that HepG2/DOX cells present distinctive small RNA profiles compared to HepG2 cells.
(TIF)

Figure S3 **Base compositions of small RNAs in HepG2 and HepG2/DOX cells.** Each color represents the small RNA tags whose first base is a certain base. In HepG2 and HepG2/DOX, most 22-nt small RNAs began with the base “U”.
(TIF)

Figure S4 **Mapping of small RNAs from HepG2 and HepG2/DOX cells onto human chromosomes, respectively.** Red lines indicate miRNAs that are located on the antisense chains of genome while the blue ones are on the sense chains.
(TIF)

Table S1 **Sequencing data of HepG2 and HepG2/DOX cells.**
(DOC)

Table S2 **Summary of known miRNAs in each sample.**
(DOC)

## References

[1] El-Serag HB, Rudolph KL (2007) Hepatocellular carcinoma: Epidemiology and molecular carcinogenesis. Gastroenterology 132: 2557–2576. 17570226 10.1053/j.gastro.2007.04.061

[2] Zheng T, Wang J, Chen X, Liu L (2010) Role of microRNA in anticancer drug resistance. Int J Cancer 126: 2–10. 19634138 10.1002/ijc.24782

[3] Harrison DJ (1995) Molecular mechanisms of drug resistance in tumours. J Pathol 175: 7–12. 7891229 10.1002/path.1711750103

[4] Ma J, Dong C, Ji C (2010) MicroRNA and drug resistance. Cancer Gene Ther 17: 523–531. 20467450 10.1038/cgt.2010.18

[5] Roberti A, La Sala D, Cinti C (2006) Multiple genetic and epigenetic interacting mechanisms contribute to clonally selection of drug-resistant tumors: Current views and new therapeutic prospective. J Cell Physiol 207: 571–581. 16250021 10.1002/jcp.20515

[6] Allen KE, Weiss GJ (2010) Resistance may not be futile: MicroRNA biomarkers for chemoresistance and potential therapeutics. Mol Cancer Ther 9: 3126–3136. 20940321 10.1158/1535-7163.MCT-10-0397

[7] Gottesman MM (2002) Mechanisms of cancer drug resistance. Annu Rev Med 53: 615–627. 11818492 10.1146/annurev.med.53.082901.103929

[8] Bartel DP (2004) Genomics, biogenesis, mechanism, and function. Cell 116: 281–297. 14744438 10.1016/s0092-8674(04)00045-5

[9] Lu J, Getz G, Miska EA, Alvarez-Saavedra E, Lamb J, et al (2005) MicroRNA expression profiles classify human cancers. Nature 435: 834–838. 15944708 10.1038/nature03702

[10] Iio A, Nakagawa Y, Hirata I, Naoe T, Akao Y (2010) Identification of non-coding RNAs embracing microRNA-143/145 cluster. Mol Cancer 9: 136. 20525177 10.1186/1476-4598-9-136 PMC2903500

[11] Pillai RS (2005) MicroRNA function: Multiple mechanisms for a tiny RNA? RNA 11: 1753–1761. 16314451 10.1261/rna.2248605 PMC1370863

[12] Nilsen TW (2007) Mechanisms of microRNA-mediated gene regulation in animal cells. Trends Genet 23: 243–249. 17368621 10.1016/j.tig.2007.02.011

[13] Vasudevan S, Tong Y, Steitz JA (2007) Switching from repression to activation: MicroRNAs can up-regulate translation. Science 318: 1931–1934. 18048652 10.1126/science.1149460

[14] Negrini M, Gramantieri L, Sabbioni S, Croce CM (2008) MicroRNA involvement in hepatocellular carcinoma. Anticancer Agents Med Chem 11: 500–521. 10.2174/187152011796011037 21554203

[15] Li W, Xie L, He X, Li J, Tu K, et al (2008) Diagnostic and prognostic implications of microRNAs in human hepatocellular carcinoma. Int J Cancer 123: 1616–1622. 18649363 10.1002/ijc.23693

[16] Caldas C, Brenton JD (2005) Sizing up miRNAs as cancer genes. Nat Med 11: 712–714. 16015356 10.1038/nm0705-712

[17] Zhang L, Huang J, Yang N, Greshock J, Megraw MS, et al (2006) MicroRNAs exhibit high frequency genomic alterations in human cancer. Proc Natl Acad Sci U S A 103: 9136–9141. 16754881 10.1073/pnas.0508889103 PMC1474008

[18] Blower PE, Verducci JS, Lin S, Zhou J, Chung JH, et al (2007) MicroRNA expression profiles for the nci-60 cancer cell panel. Mol Cancer Ther 6: 1483–1491. 17483436 10.1158/1535-7163.MCT-07-0009

[19] Liu H, D'Andrade P, Fulmer-Smentek S, Lorenzi P, Kohn KW, et al (2010) mRNA and microRNA expression profiles of the nci-60 integrated with drug activities. Mol Cancer Ther 9: 1080–1091. 20442302 10.1158/1535-7163.MCT-09-0965 PMC2879615

[20] Tomimaru Y, Eguchi H, Nagano H, Wada H, Tomokuni A, et al (2010) MicroRNA-21 induces resistance to the anti-tumour effect of interferon-alpha/5-fluorouracil in hepatocellular carcinoma cells. Br J Cancer 103: 1617–1626. 20978511 10.1038/sj.bjc.6605958 PMC2990590

[21] Tomokuni A, Eguchi H, Tomimaru Y, Wada H, Kawamoto K, et al (2011) Mir-146a suppresses the sensitivity to interferon-alpha in hepatocellular carcinoma cells. Biochem Biophys Res Commun 414: 675–680. 21982769 10.1016/j.bbrc.2011.09.124

[22] Yang X, Yin J, Yu J, Xiang Q, Liu Y, et al (2011) MiRNA-195 sensitizes human hepatocellular carcinoma cells to 5-fu by targeting bcl-w. Oncol Rep 27: 250–257. 21947305 10.3892/or.2011.1472

[23] Fornari F, Gramantieri L, Giovannini C, Veronese A, Ferracin M, et al (2009) Mir-122/cyclin g1 interaction modulates p53 activity and affects doxorubicin sensitivity of human hepatocarcinoma cells. Cancer Res 69: 5761–5767. 19584283 10.1158/0008-5472.CAN-08-4797

[24] Fornari F, Milazzo M, Chieco P, Negrini M, Calin GA, et al (2010) Mir-199a-3p regulates mtor and c-met to influence the doxorubicin sensitivity of human hepatocarcinoma cells. Cancer Res 70: 5184–5193. 20501828 10.1158/0008-5472.CAN-10-0145

[25] Xu G, Wu J, Zhou L, Chen B, Sun Z, et al (2010) Characterization of the small RNA transcriptomes of androgen dependent and independent prostate cancer cell line by deep sequencing. PLoS One 5: e15519. 21152091 10.1371/journal.pone.0015519 PMC2994876

[26] Zhou L, Chen J, Li Z, Li X, Hu X, et al (2010) Integrated profiling of microRNAs and mRNAs: MicroRNAs located on xq27.3 associate with clear cell renal cell carcinoma. PLoS One 5: e15224. 21253009 10.1371/journal.pone.0015224 PMC3013074

[27] Li R, Yu C, Li Y, Lam TW, Yiu SM, et al (2009) Soap2: An improved ultrafast tool for short read alignment. Bioinformatics 25: 1966–1967. 19497933 10.1093/bioinformatics/btp336

[28] Griffiths-Jones S, Grocock RJ, van Dongen S, Bateman A, Enright AJ (2006) Mirbase: MicroRNA sequences, targets and gene nomenclature. Nucleic Acids Res 34: D140–144. 16381832 10.1093/nar/gkj112 PMC1347474

[29] Calabrese JM, Seila AC, Yeo GW, Sharp PA (2007) RNA sequence analysis defines dicer's role in mouse embryonic stem cells. Proc Natl Acad Sci U S A 104: 18097–18102. 17989215 10.1073/pnas.0709193104 PMC2084302

[30] Osanto S, Qin Y, Buermans HP, Berkers J, Lerut E, et al (2012) Genome-wide microrna expression analysis of clear cell renal cell carcinoma by next generation deep sequencing. PLoS One 7: e38298. 22745662 10.1371/journal.pone.0038298 PMC3380046

[31] Benjamini Y, Hochberg Y (1995) Controlling the false discovery rate: a practical and powerful approach to multiple testing. J R Stat Soc Ser B 57: 289–300.

[32] John B, Enright AJ, Aravin A, Tuschl T, Sander C, et al (2004) Human microRNA targets. PLoS Biol 2: e363. 15502875 10.1371/journal.pbio.0020363 PMC521178

[33] Krek A, Grun D, Poy MN, Wolf R, Rosenberg L, et al (2005) Combinatorial microRNA target predictions. Nat Genet 37: 495–500. 15806104 10.1038/ng1536

[34] Wang X (2008) Mirdb: A microRNA target prediction and functional annotation database with a wiki interface. RNA 14: 1012–1017. 18426918 10.1261/rna.965408 PMC2390791

[35] Wang X, El Naqa IM (2008) Prediction of both conserved and nonconserved microRNA targets in animals. Bioinformatics 24: 325–332. 18048393 10.1093/bioinformatics/btm595

[36] Ashburner M, Ball CA, Blake JA, Botstein D, Butler H, et al (2000) Gene ontology: Tool for the unification of biology. The gene ontology consortium. Nat Genet 25: 25–29. 10802651 10.1038/75556 PMC3037419

[37] Kanehisa M, Araki M, Goto S, Hattori M, Hirakawa M, et al (2008) Kegg for linking genomes to life and the environment. Nucleic Acids Res 36: D480–484. 18077471 10.1093/nar/gkm882 PMC2238879

[38] Barik S (2008) An intronic microRNA silences genes that are functionally antagonistic to its host gene. Nucleic Acids Res 36: 5232–5241. 18684991 10.1093/nar/gkn513 PMC2532712

[39] Noda T, Nagano H, Takemasa I, Yoshioka S, Murakami M, et al (2009) Activation of wnt/beta-catenin signalling pathway induces chemoresistance to interferon-alpha/5-fluorouracil combination therapy for hepatocellular carcinoma. Br J Cancer 100: 1647–1658. 19401692 10.1038/sj.bjc.6605064 PMC2696759

[40] Sterpetti P, Marucci L, Candelaresi C, Toksoz D, Alpini G, et al (2006) Cell proliferation and drug resistance in hepatocellular carcinoma are modulated by rho gtpase signals. Am J Physiol Gastrointest Liver Physiol 290: G624–632. 16322093 10.1152/ajpgi.00128.2005

[41] Yip-Schneider MT, Klein PJ, Wentz SC, Zeni A, Menze A, et al (2009) Resistance to mitogen-activated protein kinase kinase (mek) inhibitors correlates with up-regulation of the mek/extracellular signal-regulated kinase pathway in hepatocellular carcinoma cells. J Pharmacol Exp Ther 329: 1063–1070. 19258520 10.1124/jpet.108.147306

[42] Li QL, Gu FM, Wang Z, Jiang JH, Yao LQ, et al (2012) Activation of pi3k/akt and mapk pathway through a pdgfrbeta-dependent feedback loop is involved in rapamycin resistance in hepatocellular carcinoma. PLoS One 7: e33379. 22428038 10.1371/journal.pone.0033379 PMC3302853

[43] Bonnas C, Specht K, Spleiss O, Froehner S, Dietmann G, et al (2012) Effects of cold ischemia and inflammatory tumor microenvironment on detection of pi3k/akt and mapk pathway activation patterns in clinical cancer samples. Int J Cancer 131: 1621–1632. 22213219 10.1002/ijc.27422

[44] Huynh H, Nguyen TT, Chow KH, Tan PH, Soo KC, et al (2003) Over-expression of the mitogen-activated protein kinase (mapk) kinase (mek)-mapk in hepatocellular carcinoma: Its role in tumor progression and apoptosis. BMC Gastroenterol 3: 19. 12906713 10.1186/1471-230X-3-19 PMC317301

[45] Gedaly R, Angulo P, Hundley J, Daily MF, Chen C, et al (2011) Pki-587 and sorafenib targeting pi3k/akt/mtor and ras/raf/mapk pathways synergistically inhibit hcc cell proliferation. J Surg Res 176: 542–548. 22261591 10.1016/j.jss.2011.10.045

[46] Gedaly R, Angulo P, Hundley J, Daily MF, Chen C, et al (2010) Pi-103 and sorafenib inhibit hepatocellular carcinoma cell proliferation by blocking ras/raf/mapk and pi3k/akt/mtor pathways. Anticancer Res 30: 4951–4958. 21187475 PMC3141822

[47] Marcucci G, Radmacher MD, Maharry K, Mrozek K, Ruppert AS, et al (2008) MicroRNA expression in cytogenetically normal acute myeloid leukemia. N Engl J Med 358: 1919–1928. 18450603 10.1056/NEJMoa074256

[48] Shi L, Cheng Z, Zhang J, Li R, Zhao P, et al (2008) Hsa-miR-181a and hsa-miR-181b function as tumor suppressors in human glioma cells. Brain Res 1236: 185–193. 18710654 10.1016/j.brainres.2008.07.085

[49] Bhattacharya SD, Garrison J, Guo H, Mi Z, Markovic J, et al (2010) Micro-RNA-181a regulates osteopontin-dependent metastatic function in hepatocellular cancer cell lines. Surgery 148: 291–297. 20576283 10.1016/j.surg.2010.05.007 PMC2905491

[50] Ji J, Yamashita T, Budhu A, Forgues M, Jia HL, et al (2009) Identification of microRNA-181 by genome-wide screening as a critical player in epcam-positive hepatic cancer stem cells. Hepatology 50: 472–480. 19585654 10.1002/hep.22989 PMC2721019

[51] Huang XH, Wang Q, Chen JS, Fu XH, Chen XL, et al (2009) Bead-based microarray analysis of microRNA expression in hepatocellular carcinoma: Mir-338 is downregulated. Hepatol Res 39: 786–794. 19473441 10.1111/j.1872-034X.2009.00502.x

[52] Huang XH, Chen JS, Wang Q, Chen XL, Wen L, et al (2011) MiR-338-3p suppresses invasion of liver cancer cell by targeting smoothened. J Pathol 225: 463–472. 21671467 10.1002/path.2877

[53] Chang J, Guo JT, Jiang D, Guo H, Taylor JM, et al (2008) Liver-specific microRNA mir-122 enhances the replication of hepatitis c virus in nonhepatic cells. J Virol 82: 8215–8223. 18550664 10.1128/JVI.02575-07 PMC2519557

[54] Chang J, Nicolas E, Marks D, Sander C, Lerro A, et al (2004) Mir-122, a mammalian liver-specific microRNA, is processed from hcr mRNA and may downregulate the high affinity cationic amino acid transporter cat-1. RNA Biol 1: 106–113. 17179747 10.4161/rna.1.2.1066

[55] Xu Y, Xia F, Ma L, Shan J, Shen J, et al (2011) MicroRNA-122 sensitizes hcc cancer cells to adriamycin and vincristine through modulating expression of mdr and inducing cell cycle arrest. Cancer Lett 310: 160–169. 21802841 10.1016/j.canlet.2011.06.027

[56] Hou J, Lin L, Zhou W, Wang Z, Ding G, et al (2011) Identification of mirnomes in human liver and hepatocellular carcinoma reveals mir-199a/b-3p as therapeutic target for hepatocellular carcinoma. Cancer Cell 19: 232–243. 21316602 10.1016/j.ccr.2011.01.001

[57] Yoon AR, Gao R, Kaul Z, Choi IK, Ryu J, et al (2011) MicroRNA-296 is enriched in cancer cells and downregulates p21waf1 mRNA expression via interaction with its 3′ untranslated region. Nucleic Acids Res 39: 8078–8091. 21724611 10.1093/nar/gkr492 PMC3185413

[58] Paillas S, Boissiere F, Bibeau F, Denouel A, Mollevi C, et al (2010) Targeting the p38 mapk pathway inhibits irinotecan resistance in colon adenocarcinoma. Cancer Res 71: 1041–1049. 21159664 10.1158/0008-5472.CAN-10-2726 PMC3304472

[59] Guo X, Ma N, Wang J, Song J, Bu X, et al (2008) Increased p38-mapk is responsible for chemotherapy resistance in human gastric cancer cells. BMC Cancer 8: 375. 19091131 10.1186/1471-2407-8-375 PMC2628930

[60] Ho R, Eggert A, Hishiki T, Minturn JE, Ikegaki N, et al (2002) Resistance to chemotherapy mediated by trkb in neuroblastomas. Cancer Res 62: 6462–6466. 12438236

[61] McCubrey JA, Steelman LS, Abrams SL, Lee JT, Chang F, et al (2006) Roles of the raf/mek/erk and pi3k/pten/akt pathways in malignant transformation and drug resistance. Adv Enzyme Regul 46: 249–279. 16854453 10.1016/j.advenzreg.2006.01.004

[62] McCubrey JA, Steelman LS, Chappell WH, Abrams SL, Wong EW, et al (2007) Roles of the raf/mek/erk pathway in cell growth, malignant transformation and drug resistance. Biochim Biophys Acta 1773: 1263–1284. 17126425 10.1016/j.bbamcr.2006.10.001 PMC2696318

[63] Wan PT, Garnett MJ, Roe SM, Lee S, Niculescu-Duvaz D, et al (2004) Mechanism of activation of the raf-erk signaling pathway by oncogenic mutations of b-raf. Cell 116: 855–867. 15035987 10.1016/s0092-8674(04)00215-6

