# Supplementary figures and images for: Genome-Wide Analysis of miRNA Signature Differentially Expressed in Doxorubicin-Resistant and Parental Human Hepatocellular Carcinoma Cell Lines
Source: PLoS One. 2013 Jan 24;8(1):e54111. doi: 10.1371/journal.pone.0054111 (PMC3554743; doi:10.1371/journal.pone.0054111)

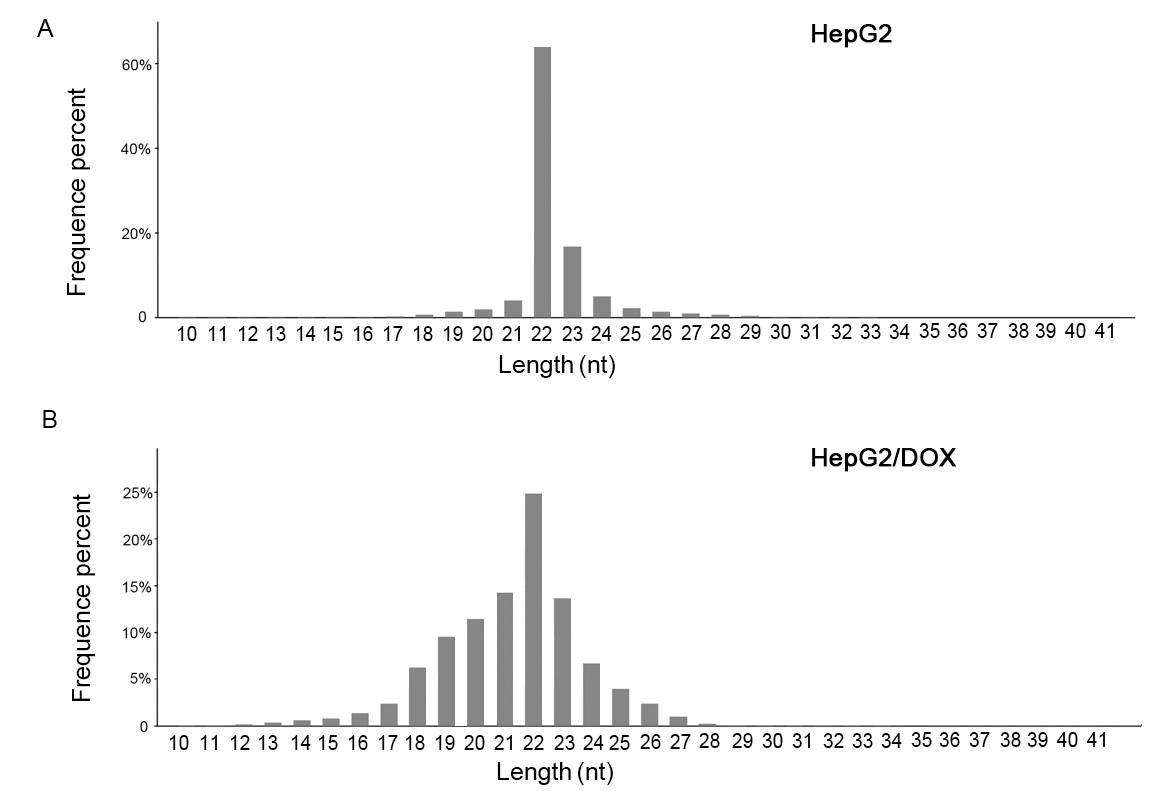

Supplement: Figure S1 — Size distributions of small RNAs in HepG2 and HepG2/DOX cells. 22-nucleotide small RNAs are enriched in both samples, which is in line with the typical size of miRNAs. (TIF) [file pone.0054111.s001.tif]

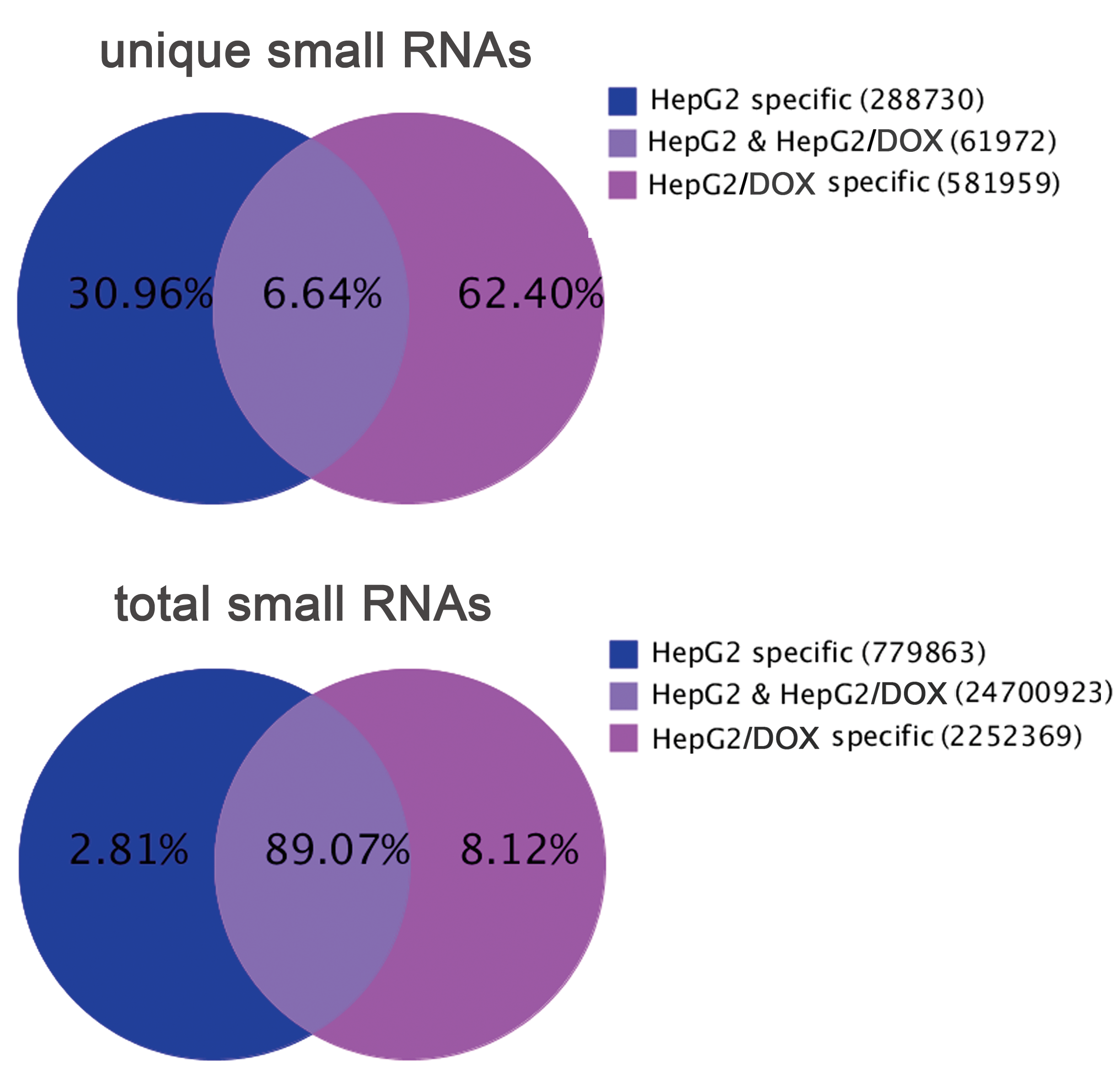

Supplement: Figure S2 — The proportions of unique common tags and total common tags of small RNAs detected in HepG2 and HepG2/DOX cells. The very few unique common tags indicate that HepG2/DOX cells present distinctive small RNA profiles compared to HepG2 cells. (TIF) [file pone.0054111.s002.tif]

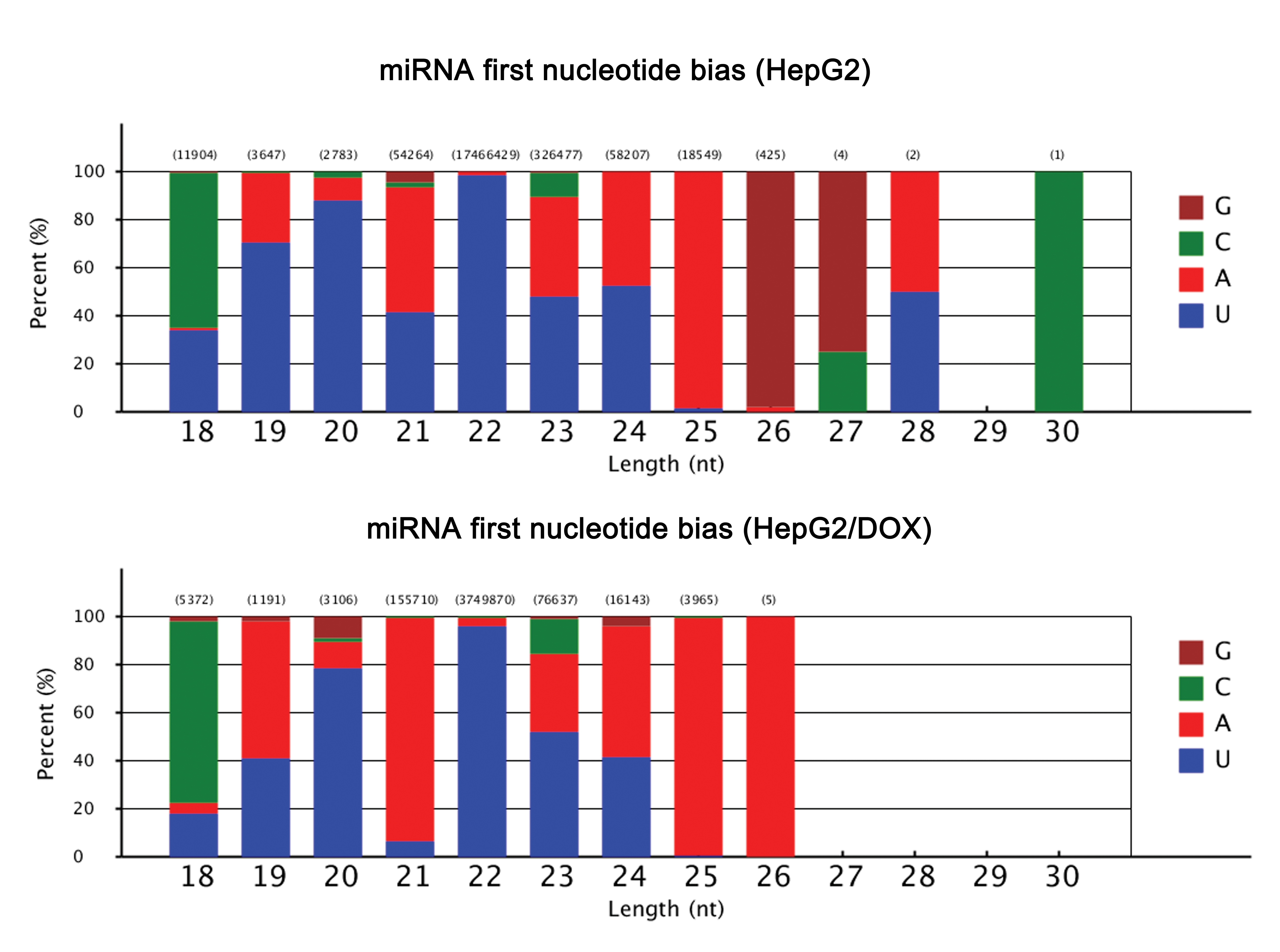

Supplement: Figure S3 — Base compositions of small RNAs in HepG2 and HepG2/DOX cells. Each color represents the small RNA tags whose first base is a certain base. In HepG2 and HepG2/DOX, most 22-nt small RNAs began with the base “U”. (TIF) [file pone.0054111.s003.tif]

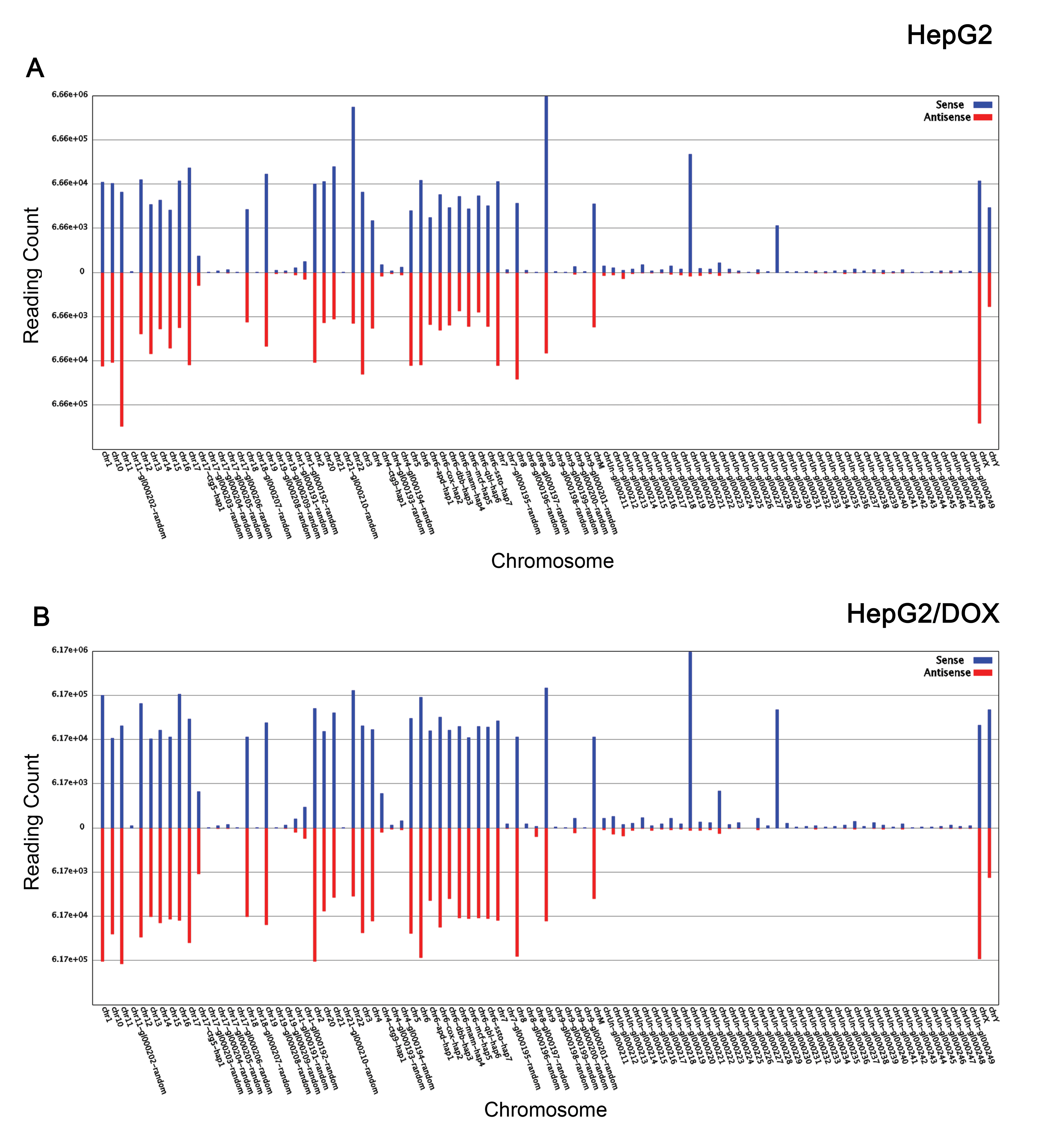

Supplement: Figure S4 — Mapping of small RNAs from HepG2 and HepG2/DOX cells onto human chromosomes, respectively. Red lines indicate miRNAs that are located on the antisense chains of genome while the blue ones are on the sense chains. (TIF) [file pone.0054111.s004.tif]
