# Supplementary material for: Genome-Wide Analysis of miRNA Signature Differentially Expressed in Doxorubicin-Resistant and Parental Human Hepatocellular Carcinoma Cell Lines
Source: PLoS One. 2013 Jan 24;8(1):e54111. doi: 10.1371/journal.pone.0054111 (PMC3554743; doi:10.1371/journal.pone.0054111)
Supplement: Table S1 — Sequencing data of HepG2 and HepG2/DOX cells. (DOC) [file pone.0054111.s005.doc]

**Table S1. Sequencing data of HepG2 and HepG2/DOX cells.**

|  | HepG2 | | HepG2/DOX | |
| --- | --- | --- | --- | --- |
| Type | Counts | Percentage (%) | Counts | Percentage (%) |
| raw reads | 14,163,632 | - | 14,653,917 | - |
| high quality reads | 14,130,291 | 100% | 14,589,505 | 100% |
| 3' adaptor-null* | 4,080 | 0.03% | 2,733 | 0.02% |
| insert-null* | 4,852 | 0.03% | 4,299 | 0.03% |
| 5' adaptor-contaminants* | 35,758 | 0.25% | 28,082 | 0.19% |
| smaller than 18nt* | 72,229 | 0.51% | 834,484 | 5.72% |
| polyA* | 95 | 0.00% | 29 | 0.00% |
| clean reads* | 14,013,277 | 99.17% | 13,719,878 | 94.04% |

Meaning of rows with * in the above table: 3' adaptor-null: number of reads with no 3' adaptor; insert-null: number of reads with no insertion; 5' adaptor-contaminants: number of 5' contaminants; smaller than 18nt: number of reads smaller than 18 nt; polyA: number of reads with polyA; clean reads: number of clean reads after adaptors and contaminants are removed.
