# Supplementary material for: Genome-Wide Analysis of miRNA Signature Differentially Expressed in Doxorubicin-Resistant and Parental Human Hepatocellular Carcinoma Cell Lines
Source: PLoS One. 2013 Jan 24;8(1):e54111. doi: 10.1371/journal.pone.0054111 (PMC3554743; doi:10.1371/journal.pone.0054111)
Supplement: Table S2 — Summary of known miRNAs in each sample. (DOC) [file pone.0054111.s006.doc]

**Table S2. Summary of known miRNAs in each sample.**

|  | miRNA | miRNA^*^ | miRNA-5p | miRNA-3p | miRNA precursors |
| --- | --- | --- | --- | --- | --- |
| Known miRNA in miRBase | 845 | 0 | 534 | 542 | 1527 |
| HepG2 | 264 | 0 | 270 | 246 | 680 |
| HepG2/DOX | 228 | 0 | 231 | 209 | 585 |
